# Development and validation of the Tuberculosis Diabetes Mellitus-Predictive Tool (TBDM-PT) in primary care

**DOI:** 10.51866/oa.708

**Published:** 2025-06-10

**Authors:** Ismail Muhammad Ikhwan, Wan Mohammad Wan Mohd Zahiruddin, Zakaria Rosnani, Abdullah Noor Hashimah, Abdullah Hasniza, Mat Jaeb Mat Zuki, Arifin Wan Nor

**Affiliations:** 1 MBChB, FAFP, FRACGP, Department of Family Medicine, School of Medical Sciences, Universiti Sains Malaysia, Kubang Kerian, Kelantan, Malaysia. Email: rosnani@usm.my; 2 MD, DrPH. EPID, Department of Community Medicine, School of Medical Sciences, Health Campus, Universiti Sains Malaysia, Kubang Kerian, Kelantan, Malaysia.; 3 MD, M. COMM. MED, Department of Community Medicine, School of Medical Sciences, Health Campus, Universiti Sains Malaysia, Kubang Kerian, Kelantan, Malaysia.; 4 MD, M. PH, Disease Control Unit, Kelantan Health Department, Kota Bharu, Kelantan, Malaysia.; 5 MD, MCM (OH), Disease Control Unit, Kelantan, Health Department, Kota Bharu, Kelantan, Malaysia.; 6 MBBS, M. MED (INT MED), Respiratory Medicine Unit, Hospital Raja Perempuan Zainab II, Kota Bharu, Kelantan, Malaysia.; 7 MBBS, MSc (Medical Statistics), PhD, (Intelligent System Techniques), Biostatistics and Research Methodology Unit, School of Medical Sciences, Health Campus, Universiti Sains Malaysia, Kubang, Kerian, Kelantan, Malaysia.

**Keywords:** Validation study, Tuberculosis, Diabetes mellitus, Screening, Primary care

## Abstract

**Introduction:**

Tuberculosis (TB) is a major public health issue worldwide. Screening patients with diabetes mellitus (DM) for TB incurs the highest cost per TB case discovered. Thus, a more practical and useful screening tool is required, especially at the primary care level. This study sought to develop and validate the Tuberculosis Diabetes Mellitus-Predictive Tool (TBDM-PT) in Kelantan, Malaysia.

**Methods:**

This study included 270 patients with DM (with and without TB) from 2019 to 2021 at health clinics in Kelantan. The variables included in the risk score were chosen using logistic regression analysis. Receiver-operating characteristic curves were used to define the cut-off points for scores indicating a low or high risk of developing TB. Nine experts and 20 healthcare workers were involved in the content and face validation processes, respectively.

**Results:**

The risk score was created using eight different variables. The cut-off point selected was 11 out of 21, with sensitivity and specificity of 81% and 90%, respectively. The content validity index of the scale was 0.93, while the face validity index of each component varied from 0.95 to 1.00.

**Conclusion:**

The newly developed TBDM-PT has great potential as a screening tool for TB among patients with DM at the primary care level. External validation and evaluation of this tool in diverse settings and larger populations are required before being applied into standard diabetes care pathways in primary care facilities.

## Introduction

In 2023, more than 1 million people died from tuberculosis (TB). Globally, it is the leading cause of death by a single infectious agent.^1^ Diabetes mellitus (DM) has been identified as a risk factor for developing TB.^2,3^ A nearly threefold increase in total deaths and deaths during TB therapy has been observed in patients with TB and DM, who have a higher mortality rate (10.3%) than those with TB alone (7.6%, P=0.001).^4^

Approximately 15% of TB cases worldwide may be linked to DM, with India and China contributing approximately 40% of these cases.^5^ Other nations across South, East and Southeast Asia also raise significant concern due to their high TB burden, the anticipated rise in DM prevalence and substantial population sizes.^5^ The prevalence of DM among patients with TB varies from approximately 5% to over 50%.^5^ Meanwhile, the prevalence of TB among individuals with DM is 1.8-9.5 times higher than that among the general population in developing Asian countries.^5^ However, there is limited evidence from studies specifically designed to address the diagnosis and management of this dual disease in these high-burden regions. Additionally, research on the potential genetic and acquired predisposition of patients with DM to TB remains scarce.^5^ A survey in Malaysia among older adult patients with DM has shown a prevalence of 1.9 per 1000 population.^6^

Screening a population for TB entails various requirements, as many cases go undetected due to their asymptomatic status and reduced health literacy.^7^ According to World Health Oganization (WHO) 2021, people with DM are at a higher risk for TB and should be considered for systematic screening.^7^ In Malaysia, in addition to symptomatic screening, chest radiography is still used as the preferred screening method for people with DM.^8^ In comparison to other at-risk groups, screening among patients with DM yields the highest cost per TB case.^8^ This is attributed to the high screening costs combined with a low yield, largely due to the use of less-than-ideal screening strategies.^8^

Worldwide, several risk scores have been developed for TB management, including active TB detection and TB survival.^9-11^ However, a TB screening trial among patients with DM using a symptomatic TB questionnaire developed by the WHO seemed to fail to detect TB effectively in this group.^10^

A practical scoring tool should be developed to enhance the detection of TB among patients with DM. This tool should identify the key factors that significantly contribute to the risk of TB in patients with DM, such as sociodemographic characteristics, TB contact history and glycaemic control, as these factors could improve the ability to predict TB.^12,13^ Therefore, this study was conducted to develop and validate a scoring tool, called the Tuberculosis Diabetes Mellitus-Predictive Tool (TBDM-PT), to predict the risk of TB among patients with DM at the primary care level in Kelantan, Malaysia.

## Methods

The study was conducted in three stages, which are explained below.

### Stage 1: Variable determination

A case control study among patients with DM was conducted in 33 primary care clinics in the Pasir Mas and Kota Bharu districts of Kelantan. Due to practical constraints in recruitment, controls were not matched to cases; however, they were demographically reasonably comparable, and potential confounding was minimised through multivariable logistic regression during analysis.

Information was acquired from patients’ medical records, the Tuberculosis Information System, the National Diabetes Registry website and interviews. Face-to-face interviews were conducted in primary healthcare clinics to complete any missing information. The study included patients with DM who had been diagnosed for at least 6 months, had no history of TB, were receiving regular care at health clinics and were aged 18 years or older. A confirmed case was defined as that with positive findings on chest radiography, sputum acid-fast bacilli test and MTB culture and sensitivity.

A family medicine specialist verified the TB diagnosis as the reference standard. Control samples were taken from all patients with DM from the same health clinics who had been screened for TB from 2019 to 2021 with similar inclusion criteria and diagnostic confirmation but were verified as negative for TB.

The Power and Sample Size Calculation software version 3.1.2 was used to determine the sample size needed based on the two-independent proportion formula for a case control design. P_0_ (0.22) was the proportion of smokers among patients with DM without TB (control), while P1 (0.09) was the expected proportion of smokers among patients with DM and TB (case).^13^ The study aimed for 80% power (fi) using a significance level (a) of 0.05, and a ratio (m) of 2 was taken, which yielded 94 and 188 patients in the control and case groups, respectively.

All data were entered into a Microsoft Excel sheet and subsequently analysed using IBM SPSS Statistics for Windows, version 26 (IBM Corp., Armonk, N.Y., USA). Simple logistic regression analysis was used to ascertain the relationship between each independent variable and TB. Variables with P-values of ≤0.25 were included for modelling in multiple logistic regression. A preliminary final model containing only variables with P-values of <0.05 was created using the forward likelihood ratio, the backward likelihood ratio and the enter variable selection techniques. Before the study’s final model was presented, the model’s fit was tested for multicollinearity, two-way interactions between variables, homogeneity of variance using the Hosmer-Lemeshow goodness-of-fit test and area under the receiver-operating characteristic (ROC) curve.

### Stage 2: Scoring development and cut-off point determination

Based on the collected data in Stage 1, the β-coefficient was determined for each significant variable. The new score was formed by dividing the coefficient values for each variable by the lowest available coefficient value for the significant variable and rounding to the closest integer.^14^ Subsequently, a value of 1 was added to all scores to represent the fact that the reference group might contribute to a score of at least 1 for the risk of TB. Based on the final score, the cut-off point was determined by using the ROC curve to differentiate between patients with high and low risks of TB for developing the TBDM-PT.

With the agreement of all research team members, the highest and leftmost point was selected as the differentiation point in the area under the curve (AUC). As screening was the main purpose of this tool, the point with the highest acceptable sensitivity (>80%) remained as the selection criteria.

### Stage 3: Content and face validation

Nine experts were selected for content validation, including three family health medicine specialists, two internal medicine specialists, two public health specialists and two medical officers in charge of the TB programme in Hospital Universiti Sains Malaysia and the primary care clinic. They were tasked with completing self-directed rating proformas containing the variables chosen for the TBDM-PT development. On a Likert scale ranging from 1 to 4, each expert was asked to rate each element, with 1 denoting that an item was not at all important to the risk score and 4 denoting that it was significant to the risk score. An answer of 1 or 2 resulted in a score of 0 for the factor, whereas an answer of 3 or 4 resulted in a score of 1 for the factor. The overall score for each component was divided by the total number of experts who replied to calculate the factor content validity index (F-CVI). The scale-level content validity index average (S-CVI/Ave) was determined by dividing the total number of experts who replied by the sum of all F-CVI. Based on the literature, the involvement of nine experts in content validation required the F-CVI and S-CVI/Ave to be at least 0.78; a value of more than 0.9 was considered to indicate excellent validity.^15,16^

Subsequently, face validation was undertaken among 20 healthcare workers (HCWs) involved in the TB screening component of the DM programmes at health clinics in Kota Bharu district, Kelantan. The HCWs included medical officers, medical assistants and staff nurses. They were asked to complete the self-directed proforma after providing consent. The following five criteria were used to determine how well participants understood the TBDM-PT: (i) the instructions given; (ii) the sentences in the risk score; (iii) the ease of scoring; (iv) the font type and size; and (v) the appropriateness of the arrangement on the TBDM-PT. On a Likert scale ranging from 1 to 4, each participant was asked to rate each component. For each mark of 3 or 4, a score of 1 was assigned, while a score of 0 was assigned for each mark of 1 or 2 for each element. The sum of the scores from each participant was used to compute the final score for each component. The face validity index (FVI) was determined by dividing the overall score by the number of participants who replied to each element. The FVI cut-off point was set at 0.83 for determining the clarity and comprehension of the TBDM-PT.^17^

## Results

A total of 270 patients with DM participated in this study, among whom 90 had TB, and 180 had no TB. This number was smaller than the planned sample size (282 participants), resulting in a reduction of the study power from 80% to 78.5%.

### Stage 1

The demographic data of the participants in Stage 1 are shown in Table 1. All participants were Malaysians. The mean participant age was 61 (SD=11) years, with 57.0% of the participants being 60 years old or above. The mean body mass index (BMI) was 26.09 kg/m^2^, with 71.5% of the sample having a BMI of more than 23 kg/m^2^. The majority of the participants with TB (72.2%) had cough symptoms for more than 2 weeks.

**Table 1:** Patient characteristics (N=270).

Outcome						
Variable	Patients with DM and TB (n=90)			Patients with DM without TB (n=180)		
	n	%	Mean (SD)	n	%	Mean (SD)
**Sex**						
Male	53	58.9		64	35.6	
Female	37	41.1		116	64.4	
**Age**			59 (11)			63 (11)
18–45 years	12	13.3		10	5.6	
46–59 years	37	41.1		57	31.7	
≥60 years	41	45.6		113	62.8	
**Marital status**						
Married	80	88.9		124	68.9	
Single/widowed	10	11.1		56	31.1	
**Number of household members**			4 (2)			4 (2)
1-4	54	60		115	63.9	
≥5	36	40		65	36.1	
**Body mass index**			23.23 (4.46)			27.48 (4.96)
<23 kg/m^2^	42	48.3		34	18.9	
≥23 kg/m^2^	45	51.7		146	81.1	
**Residential location**						
Urban	12	13.5		85	47.2	
Rural	77	86.5		95	52.8	
**Educational level**						
No formal education	7	7.8		16	8.9	
Primary education	18	20.0		30	16.7	
Secondary education	55	61.1		90	50.0	
Tertiary education (diploma or higher)	10	11.1		44	24.4	
**Presence of a BCG vaccination scar**						
Yes	84	93.3		177	98.3	
Contact with a TB patient	29	32.2		32	17.8	
History ofTB	7	7.8		7	3.9	
**Outdoor activity**			2.82 (1.46)			2.69 (1.89)
<2 h/day	9	10		59	32.8	
≥2 h/day	81	90		121	67.2	
**Income**			1369.9 (1298.7)			1232.74 (1769.4)
<RM 3030	83	92.2		158	87.8	
≥RM 3030	7	7.8		22	12.2	
**Occupation**						
Unemployed	45	50.0		97	53.9	
Healthcare worker	1	1.1		2	1.1	
Non-healthcare worker	38	42.2		48	26.7	
Pensioner	6	6.7		33	18.3	
**Duration of DM**			7.44 (5.43)			10.82 (6.62)
1-10 years	73	81.1		99	55.0	
>10 years	17	18.9		81	45.0	
**HbA1c level**			10.57 (3.02)			7.76 (1.79)
<8.0%	18	20.0		117	65.0	
≥8.0%	72	80.0		63	35.0	
DM complication	30	33.3		70	38.9	
**Type of DM treatment**						
Diet	2	2.2		10	5.6	
OAHD	46	51.1		99	55.0	
Insulin	4	4.4		9	5.0	
Combination	38	42.2		62	34.4	
Family history of DM	66	73.3		118	65.6	
Anaemia status	20	22.5		14	7.8	
**Smoking status**						
Current smoker	19	21.1		7	3.9	
Ex-smoker	2	2.2		2	2.2	
Never-smoker	69	76.7		157	87.7	
Immunosuppressive disease	11	12.2		23	12.8	
Hypertension	55	61.1		109	60.6	
**TB symptoms**						
Cough for >2 weeks	65	72.2		12	6.7	
Fever for >2 weeks	21	23.3		4	2.2	
Night sweats	16	17.8		1	0.6	
Appetite loss	26	28.9		6	3.3	
Weight loss	42	46.7		13	7.2	

DM= Diabetes Mellitus, TB= Tuberculosis, OAHD=oral anti hypoglycaemic drugs, BCG = Bacillus Calmette-Guerin

### Variable selection

Twenty-eight variables were analysed using simple logistic regression. Among them, 19 had a P-value of ≤0.25 (Appendix 1). These 19 variables were then included in the subsequent multiple logistic regression, whereby only seven variables demonstrated a significant association (Table 2).

**Table 2:** Simple and multiple logistic regression of the factors associated with TB among the patients with DM (N=270).

Variable	Crude OR (95% CI)	P-value^a^	Adjusted OR (95% CI)	P-value^b^
**SOCIODEMOGRAPHIC FACTORS**				
**Sex**				
Female	1	<0.001		0.021
Male	2.60 (1.55, 4.36)		2.78 (1.17-6.63)	
**TB HISTORY FACTORS**				
**Presence of a BCG vaccination scar**				
Yes	1	0.046		0.002
No	0.24 (0.06, 0.97)		19.76 (2.98-130.9)	
**CLINICAL AND MORBIDITY FACTORS**				
**Body mass index**				
≥23 kg/m^2^	1	<0.001		<0.001
<23 kg/m^2^	4.01 (2.28, 7.03)		7.22 (2.74-19.04)	
**DM HISTORY FACTORS**				
**Duration of DM**				
>10 years	1	<0.001		0.026
1-10 years	3.51 (1.92, 6.43)		2.90 (1.13-7.39)	
**HbA1c level**				
<8.0%	1	<0.001		<0.001
≥8.0%	7.43 (4.08, 13.54)		8.31 (3.28-21.05)	
**TB SYMPTOM FACTORS**				
**Cough for >2 weeks**				
No	1	<0.001		<0.001
Yes	36.40 (17.27, 76.71)		31.08 (11.64-82.98)	
**Fever for >2 weeks**				
No	1	<0.001		0.034
Yes	13.39 (4.44, 40.43)		6.25 (1.15-33.99)	

a Simple logistic regression

b Multiple logistic regression

No significant two-way interactions and no multicollinearity between the independent variables

Hosmer-Lemeshow test: P=0.488

Area under the curve: 0.94

Chi-square test: P=0.031

### Stage 2

*Determination of the score for each variable*

The coefficients of each significant variable from the multiple logistic regression were divided by the lowest coefficient among the variables (sex: 1.024) to obtain the score for each level of the variables, as shown in Table 3. The number was then rounded up to the nearest integer and added with 1 to indicate that at least a reference group may contribute to at least a mark of 1 for scoring.

**Table 3:** Score for each of the variables.

Predictor	β-coefficient	Divided by the lowest β-coefficienta	Nearest integer	Score (+1) mark
**Sex**				
Female	1.024	1	1	2
Male				1
**Presence of a BCG vaccination scar**				
No	2.983	2.913	3	4
Yes				1
**BMI**				
<23 kg/m^2^	1.976	1.929	2	3
≥23 kg/m^2^				1
**Duration of DM**				
1–10 years	1.063	1.038	1	2
>10 years				1
**HbA1c level**				
≥8.0%	2.118	2.068	2	3
<8.0%				1
**Cough for >2 weeks**				
Yes	3.436	3.355	3	4
No				1
**Fever for >2 weeks**				
Yes	1.833	1.790	2	3
No				1

a The lowest β-coefficient is the β-coefficient for sex (1.024)

*Detennination of the cut-off point*

**Figure 1:**
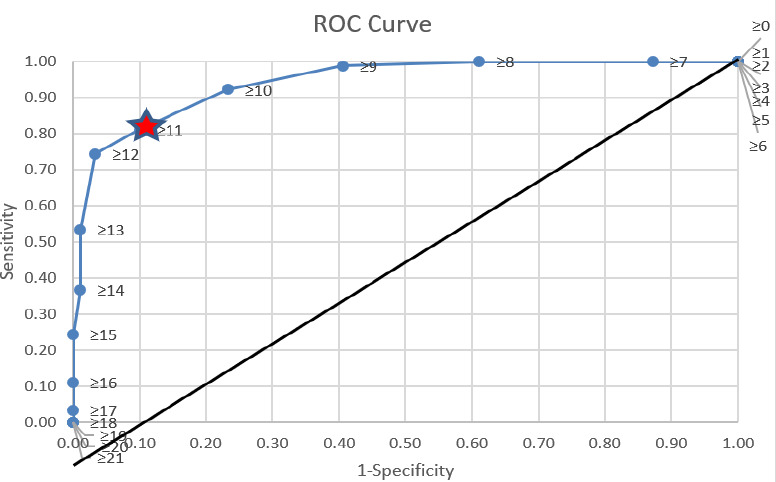
Receiver-operating characteristic curve for the cut-off point for the TBDM-PT. Score 11: Sensitivity: 81%, Specificity: 90%, AUC: 0.94

Based on the ROC curve, the cut-off point chosen for the score was 11, with a sensitivity of 81% and a specificity of 90% (Figure 1).

Based on the TBDM-PT form (Appendix 2), the risk: assessment comprised seven criteria, each assigned a score ranging from 1 to 4 according to the level of risk for developing TB. The total risk score for an individual may range from a minimum of 7 to a maximum of 21, with higher scores indicating a greater risk.

### Stage 3

*Content validation*

For content validation, all experts responded to the self-directed proforma. The majority of the validators (66.7%) were women, with the median (IQR) age being 40 (10) years and the median (IQR) experience in their field of expertise being 6 (5.0) years. The F-CVI ranged from 0.89 to 1.0, while the S-CVI/Ave was 0.93. The research team members with expertise in respiratory medicine agreed that given its clinical importance, a history of TB contact was added as a significant factor and a high-risk criterion for the TBDM-PT. The factors included in the content and face validation processes are shown in Table 4.

**Table 4:** F-CVI of each factor and S-CVI/Ave of the respondents (n=9).

Factor	Total score	F-CVI
History of TB contact	9	1.00
Sex	8	0.89
Presence of a BCG vaccination scar	8	0.89
Body mass index	8	0.89
Duration of DM	8	0.89
HbA1c level	9	1.00
Cough for >2 weeks	9	1.00
Fever for >2 weeks	8	0.89
	S-CVI/Ave	0.93

F-CVI: factor content validity index, S-CVI/Ave: scale-level content validity index average

*Face validation*

Twenty HCWs were included in the face validation process. The mean work experience was 16 (4.52) years. Approximately 60% were women, and 55% were assistant medical officers. The majority gave the TBDM-PT a mark of 4, indicating their agreement with it. To improve the readability of the TBDM-PT, the HCWs suggested to enlarge the font and rearrange the wording. The FVI of all components ranged from 0.95 to 1.00.

## Discussion

This study sought to develop and validate a TB screening tool for patients with DM. Several stages were required, which included identifying the significant variables associated with TB, developing the scoring, determining the cut-off point and assessing the face and content validity of the tool.

The factors associated with TB among the patients with DM were BMI below 23 kg/m^2^, male sex, DM duration of 10 years and below, HbA1c level above 8%, absence of a Bacillus Calmette-Guerin (BCG) vaccination scar and cough and fever persisting for >2 weeks.

This study identified a significant association between BMI of <23 kg/m^2^ and an increased risk of TB, consistent with the findings from a large-scale study conducted in China.^18^ The study examined 14,869 patients with DM for TB over 3 years; however, only 22 patients were eventually diagnosed with TB. The authors recommended targeted screening based on a low BMI, high fasting blood glucose level and low triglyceride level in detecting TB among patients with DM.^18^ Patients with both DM and TB are often underweight because rapid weight loss is a common symptom of TB. This condition is worsened by uncontrolled DM, which disrupts glucose metabolism and leads to muscle and fat breakdown. Additionally, gastroparesis, a complication of poorly managed DM, can contribute to weight loss by slowing gastric emptying.^19^

The male patients with DM in this study exhibited a higher risk of TB, aligning with prior reports on male health-seeking behaviours. Two mixed-method studies, one conducted in Uganda among 162 men and another conducted in Kelantan, Malaysia, among 381 men, highlighted poor health-seeking behaviours among this group. Delays in acknowledging health problems, seeking appropriate treatment and adhering to correct management were the main themes that emerged from these studies. These attitudes lead to delays in disease diagnosis, potentially resulting in disease progression and complications. Such health-seeking behaviours were influenced by socially constructed beliefs, demographics and external factors.^20,21^

In the current study, a DM duration of 1-10 years posed a greater TB risk than a longer disease duration. This finding might be due to early-stage challenges in managing dietary and medication regimens, leading to poor glycaemic control, as revealed by a study performed in India among 630 patients newly diagnosed with DM.^22^

Poor glycaemic control (HbA1c level of ≥8.0%) significantly elevated the TB risk, which could be explained by the fact that hyperglycaemia is linked to impaired immunity.^23,24^ Numerous studies worldwide have shown that poor glycaemic control (HbA1c level of ≥7.0%) is associated with significant morbidity and mortality with TB and other infections.^25,26^

The absence of a BCG vaccination scar also emerged as a notable risk factor in this study, corroborating findings from an Oman study, where 42.6% of patients with TB and DM lacked visible BCG vaccination scars.^27^ While the presence of a scar does not guarantee immunity, its absence may indicate reduced vaccine efficacy, warranting further immunological investigations.^27,28^

In this study, prolonged cough with or without fever was a significant TB indicator. Persistent cough is the most well-known and recognised symptom of TB.^1,7^ A study conducted in the Philippines among 4635 participants recommended TB testing for individuals with prolonged cough exceeding 2 weeks.^29^ Prolonged fever is also a well-known associated symptom of TB.^1,7^ Studies on prolonged fever and fever of unknown origin have revealed that TB is still one of the main diagnoses for this condition.^30-33^

Other TB-related symptoms include lethargy, night sweats and unexplained weight loss. However, in patients with DM and TB, the predominant symptoms are typically fever, cough or haemoptysis.^1-3^ Our study reinforces the need for enhanced TB screening among high-risk populations with DM to enable early detection and management.

Content validation ensures that a measurement tool covers all necessary aspects of what it is meant to assess.^15^ For nine experts, at least seven must agree on an item’s relevance, requiring an I-CVI of at least 0.78. In this study, the F-CVI ranged from 0.89 to 1.0. Since both the F-CVI and S-CVI/Ave exceeded 0.9, the tool demonstrated excellent content validity.^17^

Face validation evaluates whether a test appears to measure its intended purpose, using I-FVI and S-FVI-Ave with acceptable thresholds of 0.80 and 0.83, respectively. In this study, the TBDM-PT (Appendix 2) showed excellent face validity, with an I-FVI of 0.95-1.00 and an S-FVI-Ave of 0.99, indicating its clarity and suitability for TB screening in patients with DM.^15-17^ These findings suggest its potential as a new screening tool, although further research is needed to fully validate its psychometric properties.

Few studies have emphasised the importance of screening questionnaires in identifying individuals at risk, especially in resource-limited settings.^34,35^ In a study conducted in East Java, Indonesia, a TB screening tool (TB-SSR) consisting of 16 questions aimed at older adult populations was developed. The results suggested an optimal cut-off point of ≥7, with an AUC of 0.62 (P<0.001), sensitivity of 60.26% and specificity of 64.29%.^35^

A study in China found that large-scale, routine chest radiographic screenings for TB were uneconomical.^18^ It was deemed more cost-effective to focus on patients with DM at a higher risk.^18^ Similarly, a study in Sri Lanka among patients with DM at tertiary centres identified age over 45 years and the presence of a productive cough lasting 1 week or more as key criteria for active TB screening.^36^ While these two studies are comparable to our study in terms of the patient population, they did not employ a scoring system. Common risk factors such as cough, uncontrolled blood glucose levels and low BMI were significant across these studies and ours. However, our approach distinguishes itself by utilising a more specific scoring system, considering individuals with a score of 11 or higher or a history of TB contact as being at a high risk for active TB.

The TBDM-PT is a novel screening tool designed to assess TB risks in patients with DM, addressing a crucial gap, as no similar questionnaire or scoring tool has been previously reported. Developing a universal TB screening tool for patients with DM is challenging due to the heterogeneity of both diseases. While screening tools help identify high-risk individuals, they should complement, not replace, clinical judgement. Healthcare providers must remain vigilant, especially in high-burden regions, where TB can occur even without overt risk factors. A comprehensive screening approach combining the TBDM-PT, clinical evaluation and diagnostic tests is essential for optimising TB detection in patients with DM.

This study utilised multivariate analysis to identify TB risk factors in patients with DM, addressing potential confounders. However, retrospective data from 2019-2021 may limit verification due to reliance on secondary sources. Future research with primary data collection would enhance reliability.

The TBDM-PT scoring system was developed based on existing literature to ensure global relevance. The TB risk cut-off point was determined using standardised analysis to achieve the highest AUC, with validation from experts in public health, respiratory medicine, family medicine and internal medicine. However, the scoring system includes only eight criteria, excluding factors such as TB exposure duration, genetics and health literacy, which may introduce bias. Future studies incorporating additional variables and qualitative research may provide deeper insights into TB risk factors in patients with DM.

Content and face validation were conducted with expert input, supporting the TBDM-PT’s use for TB screening. However, limitations include varying expert opinions based on personal experience. The median expert experience for content validation was 6 years, which may not fully reflect broader expert consensus. Face validation, based on subjective judgement, focused on item relevance rather than deeper diagnostic utility. Future studies should include experts with at least 10 years of experience to enhance evaluation accuracy and improve clinical decision-making.

This study highlights the potential of the TBDM-PT in TB screening among patients with DM but emphasises the need for further refinement, broader validation and integration with clinical practice.

## Conclusion

The newly developed TBDM-PT has potential as a valid and reliable screening tool for TB among patients with DM in primary care settings, but it is subject to further research to confirm its comprehensive psychometric properties, including validity, reliability and sensitivity.

## Figures and Tables

**Appendix 1:** Simple logistic regression of the factors associated with TB among patients with DM attending Klinik Kesihatan in Kelantan from January 2019 to June 2021 (N=270).

Variable	Crude OR (95% CI)	P-value^a^
**SOCIODEMOGRAPHIC FACTORS**		
**Age**		
0-45 years	1	0.010
46-59 years	0.54 (0.21, 1.38)	0.200
≥60 years	0.30 (0.12, 0.75)	0.010
HbA1c level	9	1.00
**Marital status**		
Single/divorced/widowed	1	
Married	3.61 (1.74, 7.49)	
Number of household members		0.534
1-4	1	
≥5	1.18 (0.70, 1.98)	
**Outdoor duration activity**		
<2 h	1	<0.001
≥2 h	4.38 (2.06, 9.34)	
**Residential location**		
Rural	1	
Urban	0.17 (0.09, 0.34)	<0.001
**Educational level**		
None	1	0.077
Primary education	1.37 (0.47, 3.97)	0.560
Secondary education	1.39 (0.54, 3.61)	0.490
Tertiary education (diploma or higher)	0.52 (0.17, 1.60)	0.068
**Occupation**		
Unemployed	1	0.021
Healthcare worker	1.08 (0.10, 12.20)	0.952
Non-healthcare worker	1.71 (0.98, 2.97)	0.058
Pensioner	0.39 (0.15, 1.00)	0.051
**Income**		
<RM 3030 (B40)	1	0.270
≥RM 3030 (M40+T20)	0.61 (0.25, 1.48)	
**Sex**		
Female	1	<0.001
Male	2.60 (1.55, 4.36)	
**TB HISTORY FACTORS**		
**Presence of a BCG vaccination scar**		
Yes	1	0.046
No	0.24 (0.06, 0.97)	
**History of TB**		0.534
No	1	0.183
Yes	2.08 (0.71, 6.14)	
**History of TB contact**		
No	1	0.008
Yes	2.20 (1.23, 3.94)	
**CLINICAL AND MORBIDITY FACTORS**		
**Body mass index**		
≥23 kg/m^2^	1	<0.001
<23 kg/m^2^	4.01 (2.28, 7.03)	
**Anaemia**		
No	1	0.001
Yes	3.44 (1.64, 7.19)	
**Immunosuppressive disease**		
No	1	0.897
Yes	0.95 (4.41, 2.05)	
**Hypertension**		
No	1	0.930
Yes	1.02 (0.61, 1.72)	
**Smoking status**		
Non-smoker	1	<0.001
Current smoker	6.18 (2.48, 15.37)	<0.001
Ex-smoker	0.30 (0.07, 1.36)	0.120
**Alcohol consumption status**		
Never-drinker	1	0.537
Social drinker	0.48 (0.13, 1.75)	0.265
Ex-drinker	0.00 (0.00, 0.00)	1.000
**DM HISTORY FACTORS**		
**Duration of DM**		
>10 years	1	<0.001
1-10 years	3.51 (1.92, 6.43)	
**HbA1c level**		
<8.0%	1	<0.001
≥8.0%	7.43 (4.08, 13.54)	
**DM complications**		
No	1	0.373
Yes	0.79 (0.46, 1.34)	
**Family history of DM**		
No	1	0.197
Yes	1.45 (0.83, 2.53)	
**Type of DM treatment**		
Diet	1	
OAHD	2.32 (0.49, 11.03)	0.289
Insulin	2.22 (0.33, 15.18)	0.415
Combination	3.07 (0.64, 14.74)	0.162
**TB SYMPTOM FACTORS**		
**Cough for >2 weeks**		
No	1	<0.001
Yes	36.40 (17.27, 76.71)	
**Fever for >2 weeks**		
No	1	<0.001
Yes	13.39 (4.44, 40.43)	
**Night sweats**		
No	1	<0.001
Yes	38.70 (5.04, 297.16)	
Appetite loss		0.534
No	1	<0.001
Yes	11.78 (4.64, 29.94)	
**Weight loss**		
No	1	<0.001
Yes	11.24 (5.58, 22.64)	

a Simple logistic regression

No significant two-way interactions and no multicollinearity between the independent variables Hosmer–Lemeshow test: P=0.488

Area under the curve: 0.94

**Appendix 2. Tuberculosis Diabetes Mellitus Predictive Tools (TBDM-PT):**
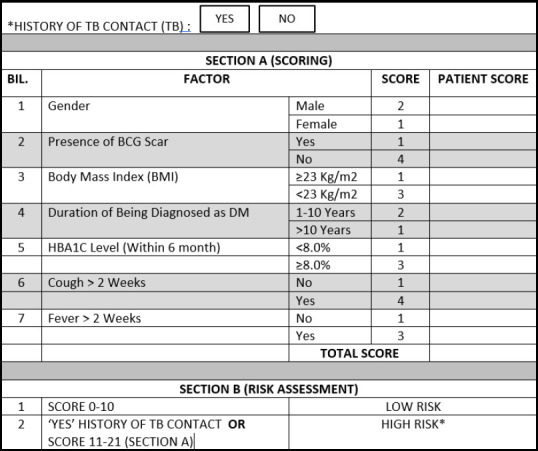


## Data sharing statement

Data are available upon request from the corresponding author.

**How does this paper make a difference in general practice?**

- The newly developed Tuberculosis Diabetes Mellitus-Predictive Tool demonstrates significant potential as a screening instrument for tuberculosis among individuals with diabetes mellitus in primary care settings. However, external validation and comprehensive evaluation across diverse populations and clinical environments are necessary prior to its integration into standard diabetes care pathways within primary healthcare facilities.
